# In vitro assessment of two novel Cellulases from *Trabulsiella odontotermitis* for agricultural waste utilization

**DOI:** 10.1186/s12896-021-00687-6

**Published:** 2021-03-23

**Authors:** Martha María Arevalos-Sánchez, Adrián Omar Maynez-Perez, Felipe A. Rodríguez-Almeida, José Alfredo Martínez-Quintana, Fidel Alejandro Sanchez-Flores, Monserrath Felix-Portillo, América Chavéz-Martínez, Myrna Elena Olvera-García, Oscar Ruiz-Barrera, Agustín Corral-Luna

**Affiliations:** 1 Facultad de Zootecnia y Ecología, Universidad Autónoma de Chihuahua, Periférico Francisco R. Almada Km 1, 31453 Chihuahua, Mexico; 2 Unidad de Secuenciación Masiva y Bioinformática, Instituto de Biotecnología, Universidad Nacional Autónoma de México, Cuernavaca, Morelos Mexico

**Keywords:** Agricultural waste, Endoglucanase, β-Glucosidase, Cellulase, Synergistic effect, Termite, *Trabulsiella odontermitis*

## Abstract

**Background:**

The production of agricultural wastes still growing as a consequence of the population growing. However, the majority of these residues are under-utilized due their chemical composition, which is mainly composed by cellulose. Actually, the search of cellulases with high efficiency to degrade this carbohydrate remains as the challenge. In the present experiment, two genes encoding an endoglucanase (EC 3.2.1.4) and β-glucosidase (EC 3.2.1.21) were overexpressed in *Escherichia coli* and their recombinant enzymes (egl-FZYE and cel-FZYE, respectively) characterized. Those genes were found in *Trabulsiella odontermitis* which was isolated from the gut of termite *Heterotermes* sp. Additionally, the capability to release sugars from agricultural wastes was evaluated in both enzymes, alone and in combination.

**Results:**

The results have shown that optimal pH was 6.0 and 6.5, reaching an activity of 1051.65 ± 47.78 and 607.80 ± 10.19 U/mg at 39 °C, for egl-FZYE and cel-FZYE, respectively. The *Km* and *Vmax* for egl-FZYE using CMC as substrate were 11.25 mg/mL and 3921.57 U/mg, respectively, whereas using Avicel were 15.39 mg/mL and 2314.81 U/mg, respectively. The *Km* and *Vmax* for cel-FZYE using Avicel as substrate were 11.49 mg/mL and 2105.26 U/mg, respectively, whereas using CMC the enzyme did not had activity. Both enzymes had effect on agricultural wastes, and their effect was improved when they were combined reaching an activity of 955.1 ± 116.1, 4016.8 ± 332 and 1124.2 ± 241 U/mg on corn stover, sorghum stover and pine sawdust, respectively.

**Conclusions:**

Both enzymes were capable of degrading agricultural wastes, and their effectiveness was improved up to 60% of glucose released when combined. In summary, the results of the study demonstrate that the recombinant enzymes exhibit characteristics that indicate their value as potential feed additives and that the enzymes could be used to enhance the degradation of cellulose in the poor-quality forage generally used in ruminant feedstuffs.

**Supplementary Information:**

The online version contains supplementary material available at 10.1186/s12896-021-00687-6.

## Background

The agricultural residues associated with crop production continue to increase globally as a consequence of rising food demand. More than 1.5 billion metric tons of agricultural residues were produced in the 1990s alone [1], and Mexico currently produces around 45 million tons per year, according to the government [2]. Most of these residues remain under-utilized, owing to their chemical composition (mainly cellulose, hemicellulose, and lignin) [3], and, thus, they are burned, representing a potential environmental risk [4]. Indeed, the compositions of such residues reduce their degradation rates and limit their inclusion in animal feedstuffs. Furthermore, even though cellulose is considered the most abundant raw material in nature and constitutes an important source of renewable energy [5], the molecule remains unavailable to most mammals that lack pre-gastric fermentation capacity, owing to the absence of glycolytic enzymes, which are needed to hydrolyze the cellulose’s predominant β-1,4 bonds [6].

In nature, cellulose digestion is achieved using enzymatic complexes that include different enzymes that work synergistically, and the widely accepted mechanism underlying the enzymatic hydrolysis of cellulose involves three types of enzymes, namely endoglucanases (endo-β-1,4-glucanase, EC 3.2.1.4), which randomly hydrolyze β-1,4 linkages, cellobiohydrolases (exo-β-1,4-glucanase, EC 3.2.1.91), which break down cellobiose units from non-reducing ends, and β-glucosidases (EC 3.2.1.21), which break down glycosyl group from the non-reducing ends of cello-oligosaccharides [7]. Interestingly, cellulose digestion is generally associated with specialized microbes, including certain bacteria, protozoa, and fungi [8], which can be either free-living organisms or components of intestinal microbiota. Additionally, some insects (e.g., termites) have also been reported to produce endogenous cellulases that enhance the digestion of cellulose by symbiotic microbes [9].

The use of cellulases is widespread, especially in animal feed [10, 11] and in the textile [12], paper [13] and biofuels [14] industries. In particular, the addition of cellulases to ruminant diets has received much attention [10, 11, 15] and several commercial preparations that include bacteria or fungi-derived enzymes are available in the market. However, the results are still variable, probably due to differences in enzyme source and mode of action, the latter of which remains unclear [16]. Thus, the search for new sources of cellulases with the capacity to degrade cellulose efficiently remains a challenge.

One interesting potential source of novel cellulases is the intestinal microbiota of certain insects, such as termites. Because such insects rely on raw materials for energy, their microbiota represent an untapped mine of enzymes that can degrade cellulose, hemicellulose, and lignin. For example, the abilities of termites to digest cellulose are dependent on symbiotic associations with intestinal microbes [17, 18]. However, relatively little is known about the specific identities and functions of species and genera found in the microflora of termite guts [19, 20]. Indeed, *Trabulsiella odontotermitis*, which possesses an interesting set of genes related to cellulose degradation, with potential biotechnological applications, was only recently isolated and sequenced [21]. Accordingly, in order to harness the potential of this resource, the objective of the present study was to overexpress, purify, and characterize two recombinant cellulases from *T. odontotermitis* isolated from termite gut to evaluate their effects on the utilization of several types of agro-industrial waste.

## Results and discussion

### Protein mass

**Fig. 1:**
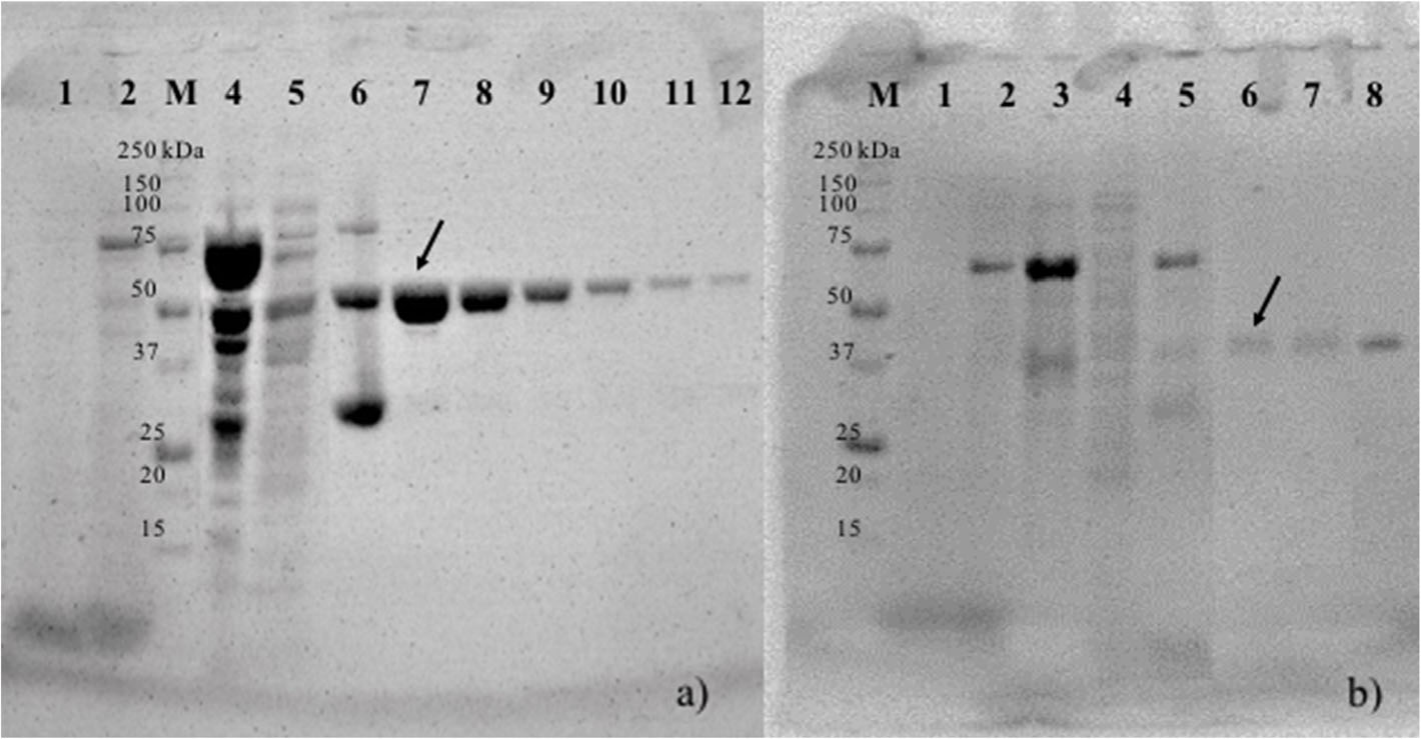
SDS-PAGE analysis of protein overexpression (**a**) recombinant egl-FZYE. Lane M: Molecular weight markers 250 kDa; Lane 1: cell extract before isopropyl-β-D-1-thiogalactopyranoside (IPTG) induction; Lane 2: cell extract after IPTG induction; Lane 4: cell extract pellet; Lane 5: cell extract supernatant; Lane 6: Resin before fusion protein cleavage reaction; Lanes 7–12: Elution of target protein (arrow). The complete picture is available in Supplementary Fig. 1. **b** recombinant cel-FZYE. Lane M: Molecular weight markers 250 kDa; Lane 1: cell extract before IPTG induction; Lane 2: cell extract after IPTG induction; Lane 3: cell extract pellet; Lane 4: cell extract supernatant; Lane 5: Resin before fusion protein cleavage reaction; Lanes 6–8: Elution of target protein (arrow). The complete picture is available in Supplementary Fig. 2

In the present study, two genes were cloned from *T. odontotermitis*, and their expressed products (egl-FZYE and cel-FZYE), which exhibited endoglucanase and a β-glucosidase activity, respectively, were selected based on previous annotation of the *T. odontotermitis* genome [21]. The estimated masses of egl-FZYE and cel-FZYE were 42 and 55 kDa, respectively, which were similar to the values predicted by the ExPASy Bioinformatics Resources Portal [22] i.e., 41.5 and 55.4 kDa, respectively), whereas the estimated masses of the egl-FZYE and cel-FZYE fusion proteins were 70 and 83 kDa, respectively (Fig. 1). Total protein recovered was 0.003 and 0.0143 mg/mL of broth for egl-FZYE and cel-FZYE.

### Protein characterization

#### Optimal pH

**Fig. 2:**
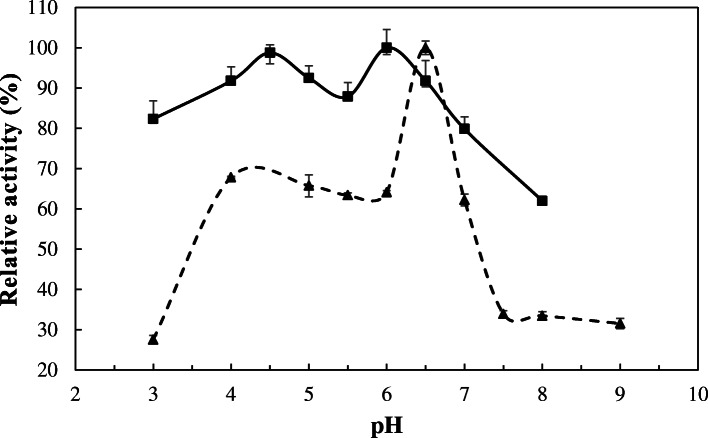
Effect of pH on the relative activity of egl-FZYE and cel-FZYE. The relative activities of egl-FZYE (squares and solid line) and cel-FZYE (triangles and hatched line) were evaluated using 1% CMC and 1% Avicel (microcrystalline cellulose), respectively

The enzyme egl-FZYE retained up to 60% of its relative activity across a wide range of pH values (3 to 8) and exhibited maximum activity at pH 6.0 (1051.65 ± 47.78 U/mg; Fig. 2). This result is similar to the optimal pH reported by Hirayama et al. [23] for NtEG, an endoglucanase that was isolated from the gut of *Nasutitermes takasagoensis*, and to those of other endoglucanases from anaerobic bacteria, which have been reported to exhibit activity over a pH range of 5.2 to 6.8 [24].

In the same way, the optimal pH for cel-FZYE was 6.5 (Fig. 2), at which it exhibited maximum activity (608.80 ± 10.19 U/mg), and the enzyme retained as much as 60% of its activity over a wide range of pH values (4 to 7). Similar results were reported by Jeng et al. [25], who evaluated three different β-glycosidases from the bacterium *Clostridium cellulovoran,* the fungus *Trichoderma reesei*, and the termite *Neotermes koshunensis*, and Scharf *el al* [26]. reported that the optimal pH range of the β-glycosidase RfBGlu-1, which was isolated from the salivary gland of *Reticulitermes flavipes*, was between 6 and 7, whereas Wang et al. [27] reported that the β-glycosidase Bgl-gs1, which was isolated from the termite *Globitermes sulphureus*, retained over 80% of its enzymatic activity over a pH range of 5 to 8 and exhibited its maximum activity at pH 6.

Interestingly, cel-FZYE exhibited strong stability under acidic conditions (pH 4.5 to 5.5), which has previously only been reported for β-glycosidases isolated from fungi (e.g., *Aspergillus niger*, *Apostichopus japonicas*, *Trichoderme resei*, and *Penicillium velutinum*) [28, 29].

#### Optimal temperature

**Fig. 3:**
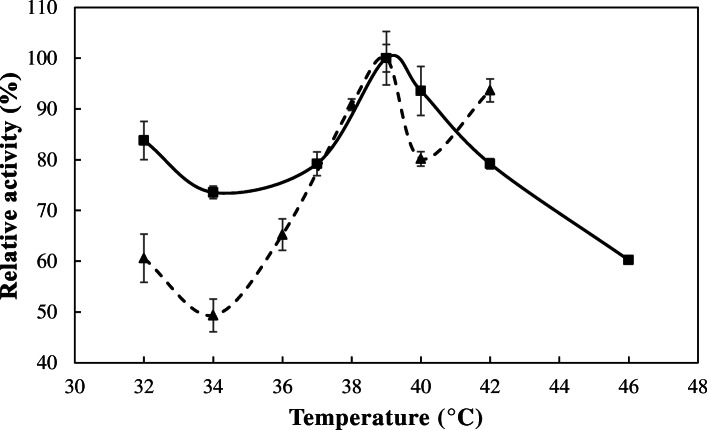
Effect of temperature on the relative activity of egl-FZYE and cel-FZYE. The relative activities of egl-FZYE (squares and solid line) and cel-FZYE (triangles and hatched line) were evaluated using 1% CMC and 1% Avicel (microcrystalline cellulose), respectively

The effect of temperature on egl-FZYE activity was evaluated using enzymatic assays, which were performed at pH 6.5. Even though this was not the optimal pH, the results obtained under these conditions are more relevant to studies of feed digestibility in ruminants, and at pH 6.5, egl-FZYE retained 91.83% of its relative activity. That being said, the optimal temperature for egl-FZYE was 39 °C, at which a specific activity of 1014.17 ± 53.71 U/mg was observed. In addition, the recombinant enzyme maintained > 60% of its relative activity from 32 to 46 °C (Fig. 3). These results are in agreement with those of *Hirayama* et al. [23], who reported the relative activities (1200 and 1392 U/mg) of two β-1,4-endoglucanases that were isolated from *Nasutitermes takasagoensis* termites and expressed in *Aspergillus oryzae* and concluded that those enzymes were highly efficient, owing to their specific activity, which was significantly higher than those that had been reported for other β-1,4-endoglucanases at that time. The specific activity of egl-FZYE was 1.74-fold higher than the activities reported for the endoglucanase RsSymEG (603 U/mg using CMC), isolated from *Reticulitermes speratus* and expressed in *Aspergillus oryzae* [30], and 34 to 204.8-fold greater than the activities of commercial enzymes isolated from *Trichoderma viride* summarized by Hirayama et al. [23]. The optimal temperature for enzymatic activity was within the range of that reported for recombinant cellulases, which is between 37 and 65 °C [31]. For example, nCfEG isolated from *Coptotermes formosanus* and expressed in *E. coli* exhibited its optimal activity at 37 °C and maintained at least 65% of its activity at 42 °C [32].

For cel-FZYE, the optimal temperature was 39 °C, at which the specific activity was 639.93 ± 17.35 U/mg (Fig. 3), and the enzyme retained ≥80% of its relative activity over a range of 38 to 42 °C. This finding is in agreement with those reported by Mattéotti et al. [33] who isolated a β-glycosidase from the intestinal microbiota of the termite *Reticulitermes santonensis*; however, that enzyme did not exhibit thermostability. In addition, cel-FZYE lost about 60% of its relative activity when the temperature was increased to 46 °C, and the optimal temperature observed for cel-FZYE corresponds to that reported for mesophyll environments, probably because it was isolated from a bacterium that inhabits the digestive tract of termites, which normally do not reach 40 °C. Furthermore, this optimal temperature is similar to those reported for β-glycosidases isolated from facultative bacteria (e.g., *Pectobacterium carotovorum* subsp. *carotovorum* and *Bacillus subtillis*), which exhibit optimal temperatures in the range of 37 to 45 °C [34, 35].

Finally, a very interesting behavior regarding the effect of pH and temperature on relative activity of the enzymes was observed. As can be observed in Figs. 2 and 3, the relative activity of egl-FZYE was decreased around 10% when pH was increased from 4.5 to 5.5 and cel-FZYE reduces their relative activity by much as 20% when temperature increase from 39 to 40 °C. However, in both cases the effect is reversible. This behavior could be explained by nonstructural conformational changes in the active site of the proteins caused for low variations in pH and temperature. Since the observed effect is reversible, it could be assumed that it might be the effect of conformational variations in the surrounding residues, particularly in those involved in recognition of the substrate, which can explain the interference with the catalytic reaction. These assumptions are based on the observations made by Moracci et al. [36] and Cairns & Esen, [37]. They reported that the Glycosyl hydrolases belonging to family 1 display a (α/β)_8_ barrel structural fold in which two glutamate residues are involved in the catalytic reaction. One of these highly conserved glutamate residue act as nucleophile and the other one as an acid/base. As reported by Moracci et al. [36] the catalytic reaction can be reduced when the acid/base glutamate residue is replaced (changed by mutation), however activity of the enzyme is not detected when the nucleophile glutamate is replaced. Moreover, crystal structure analysis of a β-glucosidase from *Bacillus polymyxa* conducted by Sanz-Aparicio et al. [38] revealed a set of determinant residues including Gln20, His121, Tyr296, Glu405 and Trp406 that are involved in substrate recognition. More interesting, small chemical changes in those residues can affect affinity to the substrate without affecting the conformational structure of the complete enzyme. Particularly, those authors reported a two bidentate hydrogen bonds made by Gln20 and Glu405 that could conform the structural explanation to reverse the inhibitor effect by aldolactones to β-glucosidases.

#### Enzyme kinetics

**Fig. 4:**
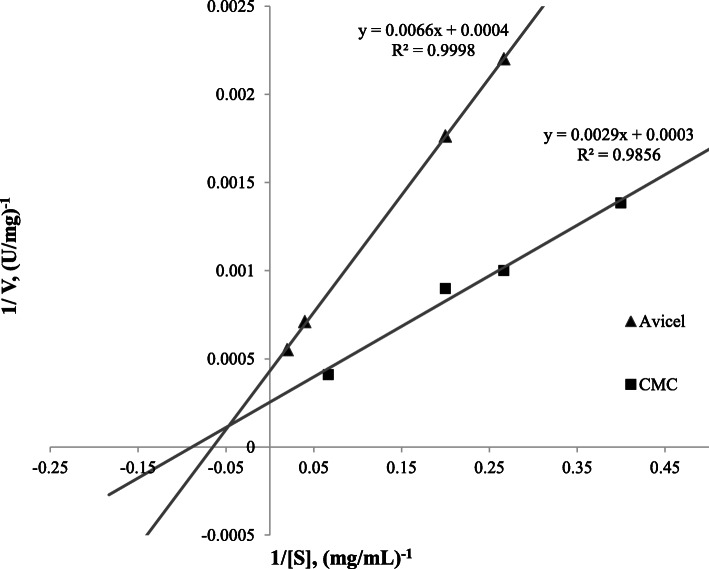
Kinetic analysis of egl-FZYE toward several concentrations of CMC and Avicel

**Fig. 5:**
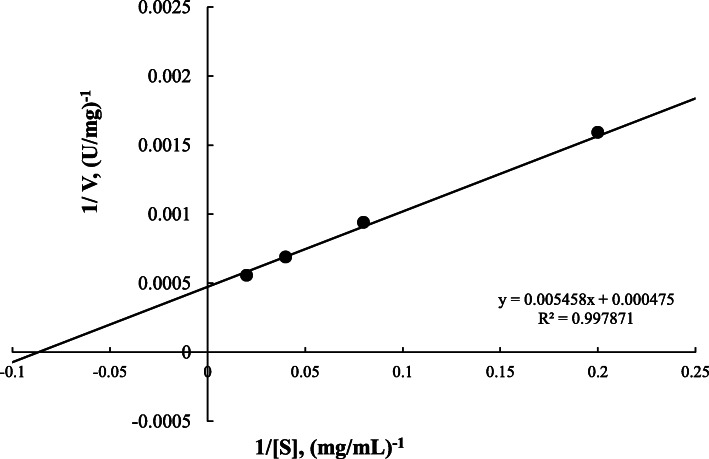
Kinetic analysis of cel-FZYE toward several concentrations of Avicel

The kinetic constants for egl-FZYE in several concentrations of CMC and Avicel were calculated using Lineweaver-Burk plots (Figs. 4 and 5). The *Km* and *Vmax* values were 11.25 mg/ml and 3921.57 U/mg, respectively, when CMC was used as substrate, and 15.39 mg/ml and 2314.81 U/mg, respectively, when Avicel was used as substrate (Fig. 4). The values obtained using CMC were 1.28- and 1.76-fold higher, respectively, than those reported for a native endoglucanase isolated from *Nasutitermes takasagoensis* [24], and even greater differences were observed between egl-FZYE and enzymes isolates that yielded values of only 2–4.67 mg/mL [30].

In the present study, egl-FZYE exhibited greater specific activity (~ 1.8-fold higher) when CMC was used as the substrate than when Avicel was used as the substrate. However, in terms of kinetic constants, the *Km* value was lower when using CMC as the substrate. This could be interpreted as a higher affinity of egl-FZYE for CMC. According to previous studies, endoglucanases randomly break down glycosidic chains, owing to a preference for amorphous regions, and therefore, insoluble crystalline cellulose (e.g., Avicel), which is highly polymerized, would not be a suitable substrate [39]. However, even in crystalline cellulose, it is possible that there are amorphous regions that can be hydrolyzed by endoglucanases [9] and that could explain the behavior of egl-FZYE in those substrates. Despite the marked differences, which can be explained by differences in chemical properties of the substrates, these findings are in agreement with those of Zhang and Lynd [40], who reported that some endoglucanases are capable of releasing considerable amounts of reducing sugar form Avicel.

Meanwhile, cel-FZYE failed to exhibit any important activity when CMC was used as the substrate but yielded *Km* and *Vmax* values of 11.49 mg/ml and 2105.26 U/mg, respectively, when Avicel was used as the substrate. As mentioned above, cel-FZYE belongs to the *β-D-*glucosidase family, members of which exhibit preferences for soluble cellobiose and other cellodextrins with degree of polymerization (DP) values of up to 6, whereas CMC possesses a DP value of 100–2000 [7].

### Effectiveness of egl-FZYE and cel-FZYE

Both enzymes exhibited activity when incubated with the agricultural wastes (Table 1). However, markedly higher activities (≤2297.7 ± 270.1 U/mg) were observed when sorghum stover was used as the substrate for egl-FZYE(*P* < 0.05), and those levels were equivalent to 24.9 ± 2.9 μM glucose *per* min, which is higher than the equivalent activities observed for corn stover and pine sawdust (6.32 ± 0.6 and 5.61 ± 2.0 μM glucose *per* min, respectively) and also greater than the activities observed with the commercial substrates (CMC and Avicel; Table 1).

**Table 1:** Effect of recombinant enzymes on several agro-industrial wastes

Substrate	Enzymatic activity (U/mg)			DSE
	egl-FZYE	cel-FZYE	50:50 mixture	
CMC	1073.9 ± 42.4^a^	642.6 ± 0.0^a^	1711.0 ± 72.8^a^	0.99
Avicel	958.6 ± 84.9^ab^	188.0 ± 30.3^b^	1346.9 ± 24.2^ab^	1.17
Corn stover	583.9 ± 56.7^bc^	278.3 ± 41.2^b^	955.1 ± 116.1^b^	1.10
Sorghum stover	2297.7 ± 270.1^d^	1042.4 ± 100^c^	4016.8 ± 332^c^	1.20
Pine sawdust	518.4 ± 193^c^	513.0 ± 66.1^ad^	1124.2 ± 241^b^	1.08

*CMC* Carboxymethyl-cellulose, *DSE* Degree of synergistic effect

These results can likely be attributed to the chemical compositions of the substrates since corn stover and pine sawdust contain around 21 and 55% lignin, respectively, which would reduce the accessibility of the enzymes to lignocellulosic materials [41], whereas sorghum stover contains only 11% lignin. In addition, previous studies have also reported that accessibility is even more important than crystallinity index in determining cellulose hydrolysis rate [42]. Furthermore, the total glucose released from sorghum stover was considerably higher than previously reported by Hess et al. [43] for the biofuel feedstocks *Miscanthus* and Switchgrass, whereas the amounts of glucose released from the corn stover and pine sawdust were relatively similar.

The enzymatic activity of egl-FZYE upon CMC and Avicel was as expected; indeed, even though some endoglucanases exhibit considerable activity for Avicel [33], CMC is a soluble substrate and, therefore, generally more suitable for digestion by endoglucanases [7].

Regarding cel-FZYE, the enzyme had catalytic activity upon both sorgum stover and pine sawdust (Table 1), being the last one comparable to the one observed for egl-FZYE. The lesser activity of cel-FZYE on all substrates tested for -when compared to that of egl-FZYE- could be due to its own nature; it is known that β-glucosidases hydrolyze soluble cellobiose and other cellodextrins with DP values of up to 6 [7]. However, the enzymatic activities decrease with increasing DP. The DP values of Avicel and CMC could be between 500 and 2000, respectively, whereas those of agricultural wastes are expected to be over 2000.

**Fig. 6:**
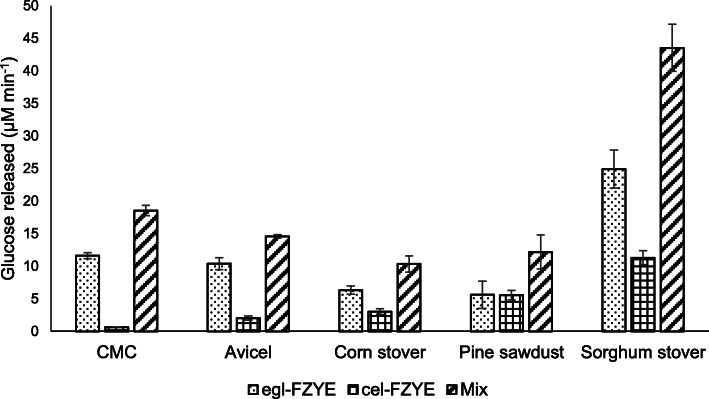
Effectiveness of individual and mixed enzymes in the digestion of agro-industrial wastes, CMC and Avicel

Finally, the DSE values clearly indicated that the enzymes exhibited an important synergistic effect (Table 1). The enzyme mixture released 40–116% more total reducing sugar from all the substrates than the individual enzymes (*P* < 0.05; Fig. 6). Similarly, high levels of synergistic effect (≤50.6%) have also been reported for cellulases and β-glucosidases of the fungus *Trichoderma reesei* isolated from different sources and at different concentrations [44]. The main contributions of β-glucosidases to cellulose degradation are the production of reducing sugars from short, beta-linked oligosaccharides, which are the products released by endoglucanases and exoglucanases, and the reduction of enzymatic inhibition caused by the production of their own end products [45]. These results indicate that this mixture of enzymes has a wide range of applications. Indeed, the amounts of glucose released by the 50:50 mixture from corn and sorghum stover were five- and 20-fold higher than those released by 13 recombinant endoglucanases from CMC and 12- to 51-fold higher than those released by the same 13 endoglucanases from alfalfa hay [46].

## Conclusions

Both of the purified enzymes evaluated in the present study clearly exhibited cellulase activity and were capable of releasing considerable amounts of glucose from agricultural wastes (corn and sorghum stover), as well as from pine sawdust. The activity of egl-FZYE was greater than that of cel-FZYE. However, the activity was significantly improved when the enzymes were used together, thereby revealing an important synergistic effect. The optimal pH and temperature conditions for the enzymes, either alone or in combination, were very similar to the physiological conditions of ruminal environments. Therefore, these findings confirm the potential value of egl-FZYE and cel-FZYE for use as a feed additive for improving fiber digestibility and for facilitating increases in the amount of corn and sorghum stover included in ruminant feed.

## Methods

### Synthetic genes and vector constructs

The sequences of the endoglucanase and ß-glucosidase genes were obtained from the genome annotation analyses performed for five *T. odontotermitis* strains isolated from the digestive tract of the termite *Heterotermes* sp. [21]. The coding sequences of both genes (omitting stop codons) were synthesized carrying the restriction sites for the NdeI and SapI enzymes at the 5′ and 3′ ends, respectively. Each construct was then cloned into the commercial IMPACT vector pTXB1 (New England Biolabs, Ipswich, MA, USA) by GenScript (Piscataway, NJ, USA), which has a chitin-binding domain (CBD) that allows for affinity purification of the recombinant protein on a chitin resin column. In addition to all the common features in expression vectors, pTXB1 also has an intein splicing element that -in the final construct- has the target gene at the N-terminus and the CBD at the C-terminus. Upon on-column induction, the intein undergoes self-cleavage, releasing the target protein without any extra amino acids in a single chromatographic step [47–50]. We called the 1104 bp endoglucanase-harboring vector pTXB1-*EG-FZYE* and 1443 bp ß-glucosiodase-harboring vector pTXB1-*CEL-FZYE*. Both gene sequences were deposited in the database of the National Center for Biotechnology Information under the accession nos. MW013833 and MW013834 for the endoglucanase and β-glucosidase*,* respectively.

### Expression and purification of recombinant proteins

Competent cells of *E. coli* strain BL21 (obtained from Sigma-Aldrich) were transformed using the expression vectors (pTXB1-*EGL-FZYE* and pTXB1-*CEL-FZYE*) and a previously described heat shock procedure [51] and then grown in 100 mL of LB, with ampicillin (100 μg/mL), at 37 °C under constant shaking (180 rpm). When the OD_600_ of the cultures reached 0.5–0.6, expression of the recombinant proteins was induced by the addition of 0.2 mM isopropyl-β-D-1-thiogalactopyranoside (IPTG), after which the cultures were incubated for an additional 5 h at 30 °C and then harvested by centrifugation (5810R; Eppendorf, Hamburg, Germany) at 3220×*g* for 20 min at 4 °C. After removal of the supernatant, the cell pellets were re-suspended in a volume of Column Buffer corresponding to 10% of the original culture volume (20 mM Tris-HCl, pH 8.5 (500 mM NaCl, 1 mM EDTA, 0.25% Triton), kept on ice, and disrupted by ultrasonic treatment (UP400St; Hielscher Ultrasonics, Teltow, Germany), using eight rounds of 10 s at 20 W and 10 s of incubation on ice each. The lysate was centrifuged at 15,000×*g* for 30 min at 4 °C, and the supernatant was recovered. The recombinant proteins were then purified using affinity chitin columns (New England Biolabs, Massachusetts, US) previously equilibrated using column buffer (20 mM Tris-HCl, 500 mM NaCl, 1 mM EDTA, 0.25% Triton, pH 8.5). To pack the column one volume of chitin resin was used per each10 volumes of supernatant. After loading the supernatant, the column was washed using 20 mL of column buffer to remove unbound proteins, and intein cleavage was induced by quickly flushing the column with 3 mL cleavage buffer (column buffer containing 50 mM DTT). After the cleavage buffer was evenly distributed throughout the column, it was incubated for 40 h at 4 °C, and the target protein was eluted from the column using 5 mL column buffer. Fractions (1 mL each) were collected from the column and then analyzed using 12% SDS-PAGE (Mini-PROTEAN TGX Stain-Free Gels; BioRad, Hercules, CA. USA), and the protein concentration was determined using the Bradford method, with bovine serum albumin as a standard [52].

### Enzymatic activity

The enzymatic activity of the recombinant proteins was determined using the 3,5-dinitrosalicylic acid (DNS) method [53]. The enzymatic reaction mixture (300 μL) was prepared using equal volumes of enzyme (0.06 mg/mL) and carboxymethyl cellulose CMC reagent (1% w/v; Golden Bell Reactivos, Mexico City, Mexico), which was diluted using phosphate buffer (0.05 M, pH 7), and then incubated for 1 h at 39 °C with slow agitation (80 rpm). The reaction was stopped by adding DNS reagent (1.4% NaOH, 0.75% 3–5 dinitrosalicylic acid, 10% sodium and potassium tartrate, 0.54% phenol, and 0.59% sodium metabisulfite) and boiling the mixture for 3 min and immediately transferred to ice bath to stop the reaction. Aliquots (100 μL) of the boiled samples were transferred to a 96-well microplate (Corning, New York, NY, US). The assays were conducted in duplicate and measured at 540 nm using a microplate spectrophotometer (Multiskan GO; Thermo Scientific, Waltham, MA, USA). The amount of glucose released was quantified using a standard curve (50–5000 μM glucose). One unit (U) of endoglucanase or β-glycosidase activity was defined as the amount of enzyme producing 1 μmol of glucose per minute, and the specific activity of each enzyme was expressed as U/mg protein.

### Characterization of recombinant proteins

To determine the optimal pH of the enzymes, CMC (1% w/v) was dissolved in phosphate buffer (0.05 M) that had been adjusted to different pH values (3.0, 4.0, 4.5, 5.0, 5.5, 6.0, 6.5, 7.0, or 8.0), and enzyme activity was measured using the same reaction mixtures and conditions described above. To determine the optimal temperature of the enzymes, enzyme activity (relative activity at the optimal pH) was measured at different temperatures (32, 34, 37, 39, 40, 42, or 46 °C).

The kinetic parameters were determined according to the Michaelis-Menten constants, *Km* and *Vmax*, which were determined using CMC (2.50, 3.75, 5, or 15 mg/mL) and Avicel (microcrystalline cellulose; 3.75, 5, 12.50, 25, or 50 mg/mL) as substrates, and the reaction mixtures were prepared as described above, using optimal pH and temperature conditions (pH 6.5 and 39 °C) were used. The data were plotted according to the Lineweaver-Burk method [54].

### Effect of egl-FZYE and cel-FZYE on agricultural wastes

The abilities of the enzymes to release glucose from different agricultural wastes was evaluated using the DNS method, as previously described by [53]. Corn and sorghum stover were dried and milled at 0.5 mm and pine sawdust was used as a reference raw material. The substrates were diluted in phosphate buffer (50 mM, pH 6.5), and the reactions were performed as described above, using optimal pH and temperature (pH 6.5 and 39 °C, respectively) and 0.06 mg/mL of either enzyme.

Meanwhile, to investigate the synergy of the enzymes, a 50:50 mixture of egl-FZYE and cel-FZYE was evaluated for its ability to convert CMC, Avicel, corn stover, sorghum stover, and pine sawdust into glucose. Reactions were performed as described above, using 0.06 mg/mL of the enzyme mixture. The substrates were prepared at 1% w/v in phosphate buffer (50 mM, pH 6.5) and incubated for 1 h at 39 °C with slow agitation (80 rpm). The process was then repeated using each enzyme individually, and the degree of synergistic effect (DSE) of the binary mixture was calculated as follows: DSE = GC_(Mixture)_ / (GC_(egl-FZYE)_ + GC_(cel-FZYE)_), where the GC values represent the amounts of glucose produced by the enzyme mixture, egl-FZYE, and cel-FZYE, respectively [55].

### Data analysis

All experiments were performed in triplicate. Data were subjected to analysis of variance (ANOVA), and mean values were compared by using Tukey HSD test. All analyses were conducted using SAS 9.0 (SAS Institute, Inc., Cary, NC, USA), and statistical significance was defined as *P* < 0.05.

## Supplementary Information

**Additional file 1.**

**Additional file 2.**

## Data Availability

The datasets generated and/or analyzed during the current study are not publicly available due these are subject to protection through patent, but are available from the corresponding author on reasonable request*.* Both gene sequences were deposited in the database of the National Center for Biotechnology Information under the accession nos. MW013833 (permanent link: https://www.ncbi.nlm.nih.gov/nuccore/MW013833) and MW013834 (permanent link: https://www.ncbi.nlm.nih.gov/nuccore/MW013834) for the endoglucanase and β-glucosidase*,* respectively.
