# Supplementary material for: In vitro assessment of two novel Cellulases from Trabulsiella odontotermitis for agricultural waste utilization
Source: BMC Biotechnol. 2021 Mar 23;21:26. doi: 10.1186/s12896-021-00687-6 (PMC7986525; doi:10.1186/s12896-021-00687-6)
Supplement: Supplementary file 1 — Additional file 1. [file 12896_2021_687_MOESM1_ESM.docx]

***In vitro* Assessment of Two Novel Cellulases from *Trabulsiella odontotermitis* for Agricultural Waste Utilization**

Martha María Arevalos-Sánchez^a^, Adrián Omar Maynez-Perez^a^, Felipe A. Rodríguez-Almeida^a^, José Alfredo Martínez-Quintana^a^, Fidel Alejandro Sanchez-Flores^b^, Monserrath Felix-Portillo^a^, América Chavéz-Martínez^a^, Myrna Elena Olvera-García^b^, Oscar Ruiz-Barrera^a^ and Agustín Corral-Luna^a^*

^a^ Facultad de Zootecnia y Ecología, Universidad Autónoma de Chihuahua. Periférico Francisco R. Almada Km 1, Chihuahua, Chihuahua, 31453, México.

^b^ Unidad de Secuenciación Masiva y Bioinformática, Instituto de Biotecnología, Universidad Nacional Autónoma de México, Cuernavaca, Morelos, México.

* Corresponding author

E-mail: acorral@uach.mx (Agustín Corral-Luna)

RAW IMAGES


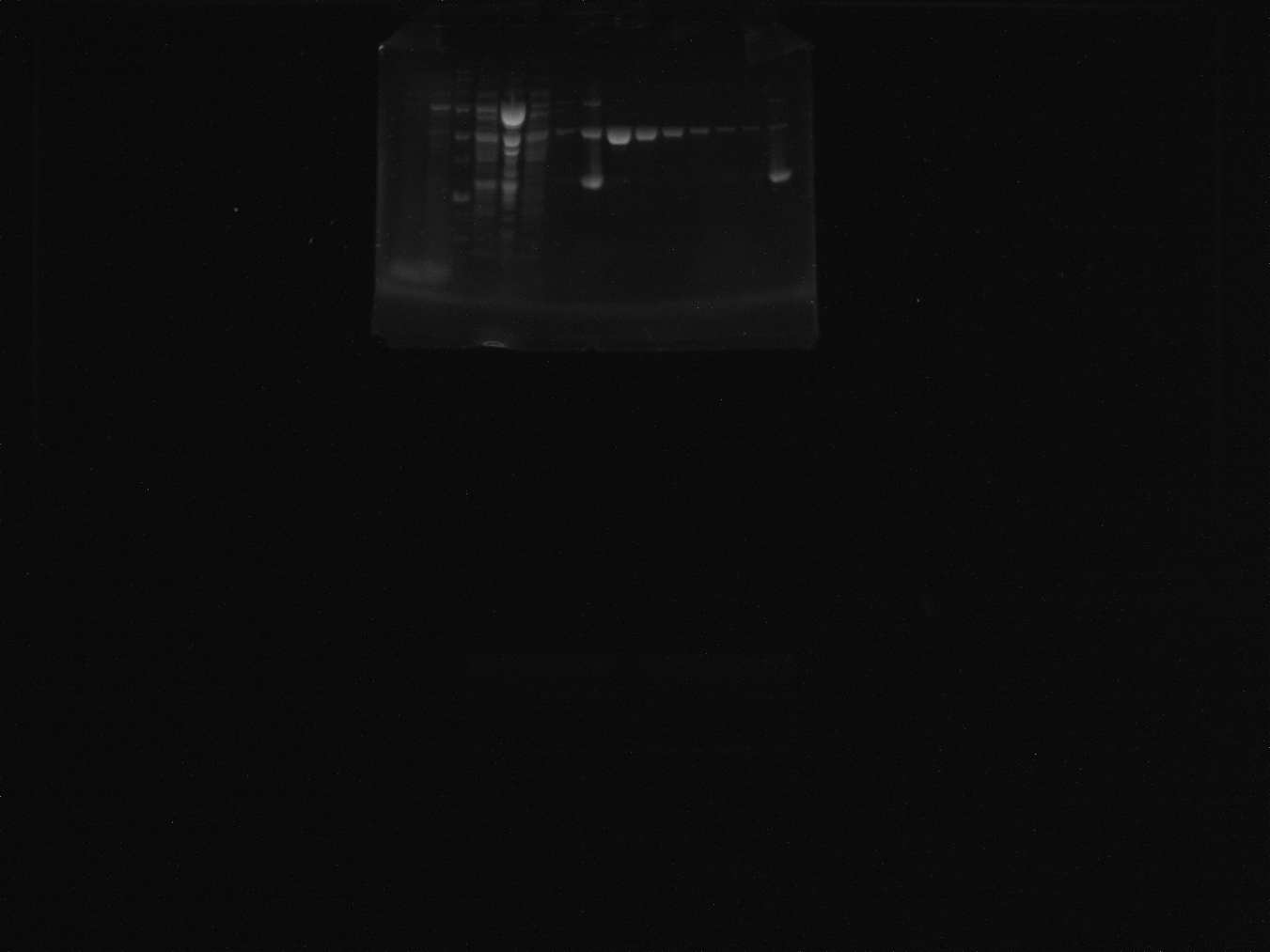


This is the raw image taken from the SDS-PAGE gel presented in the Figure 1a. The colors of the gel were inverted (color black to white) to enhance the visualization of the bands.

In addition, two intermediate lanes (before the lanes that showed the purified protein) were eliminate because the aliquots in those lanes involved the protein still embedded in the silica before separation and were not important for the presentation of the results in the manuscript.


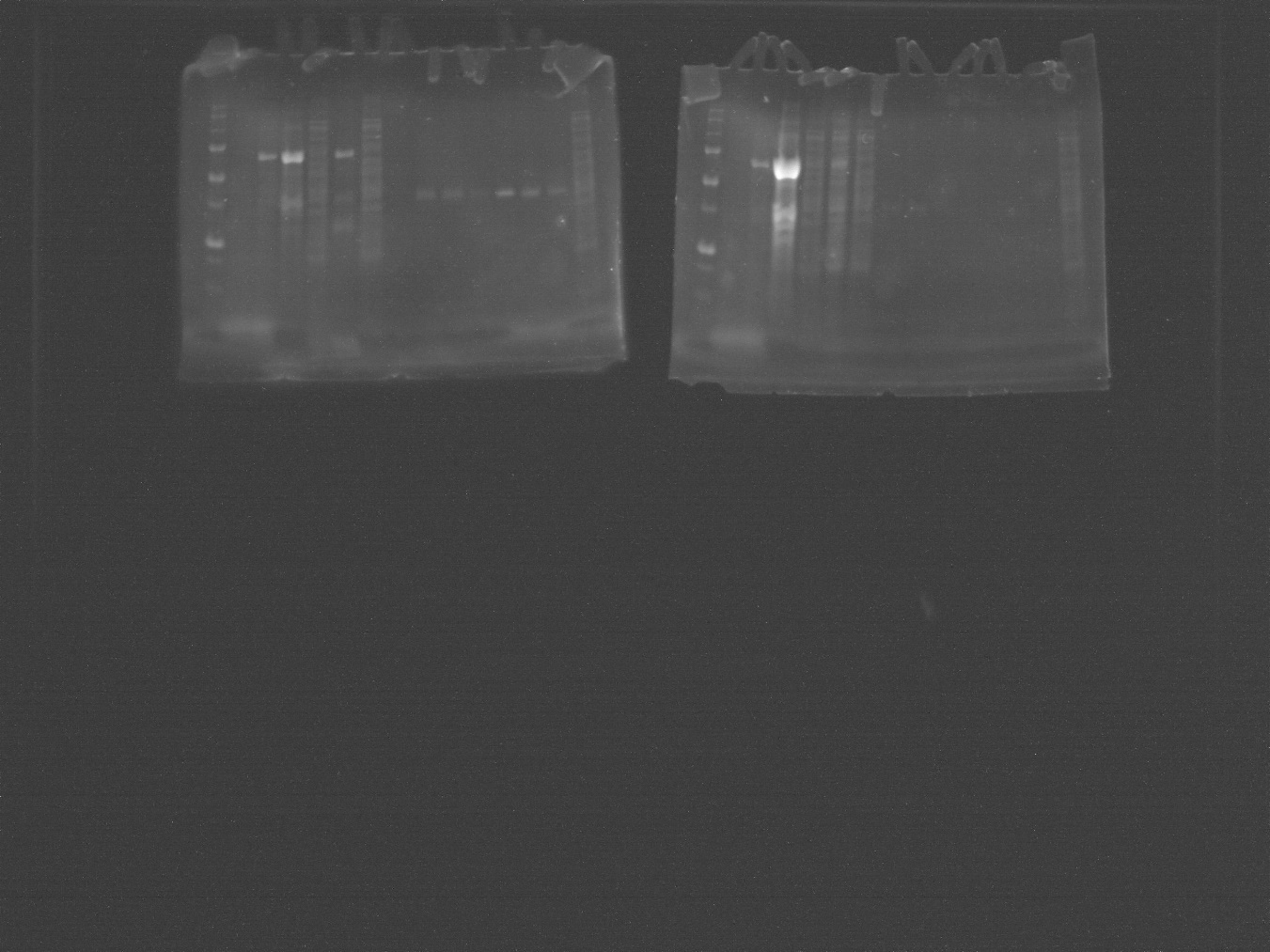


This is the raw image taken from the SDS-PAGE gel to present in the Figure 1b. Firstly the image was cropped to eliminate the gel from the right that was a different part of the project.

The gel that is in the left of the image was used for the manuscript (Fig 1b). The colors of the gel were inverted (color black to white) to enhance the visualization of the bands. Also, the last 4 lanes of the gel were cut and eliminate because we did not saw noteworthy difference after the 3 first fractions of the purified protein. Finally, two intermediate lanes (before the lanes that showed the purified protein) were eliminate because the first did not have enough definition and the second was empty, this can be due to an error on experimentation since those lanes involved the protein embedded in the silica before separation, moreover those aliquots were not of great importance for the presentation of the results in the manuscript.
