# Supplementary material for: In vitro assessment of two novel Cellulases from Trabulsiella odontotermitis for agricultural waste utilization
Source: BMC Biotechnol. 2021 Mar 23;21:26. doi: 10.1186/s12896-021-00687-6 (PMC7986525; doi:10.1186/s12896-021-00687-6)
Supplement: Supplementary file 2 — Additional file 2. [file 12896_2021_687_MOESM2_ESM.docx]

***In vitro* Assessment of Two Novel Cellulases from *Trabulsiella odontotermitis* for Agricultural Waste Utilization**

Martha María Arevalos-Sánchez^a^, Adrián Omar Maynez-Perez^a^, Felipe A. Rodríguez-Almeida^a^, José Alfredo Martínez-Quintana^a^, Fidel Alejandro Sanchez-Flores^b^, Monserrath Felix-Portillo^a^, América Chavéz-Martínez^a^, Myrna Elena Olvera-García^b^, Oscar Ruiz-Barrera^a^ and Agustín Corral-Luna^a^*

^a^ Facultad de Zootecnia y Ecología, Universidad Autónoma de Chihuahua. Periférico Francisco R. Almada Km 1, Chihuahua, Chihuahua, 31453, México.

^b^ Unidad de Secuenciación Masiva y Bioinformática, Instituto de Biotecnología, Universidad Nacional Autónoma de México, Cuernavaca, Morelos, México.

* Corresponding author

E-mail: acorral@uach.mx (Agustín Corral-Luna)

RAW IMAGES

**1 2 M A 4 5 B 6 7 8 9 10 11 12 C**

**250 KDa**

**100**

**50**

**37**

**25**

**15**

**150**

**75**

**20**


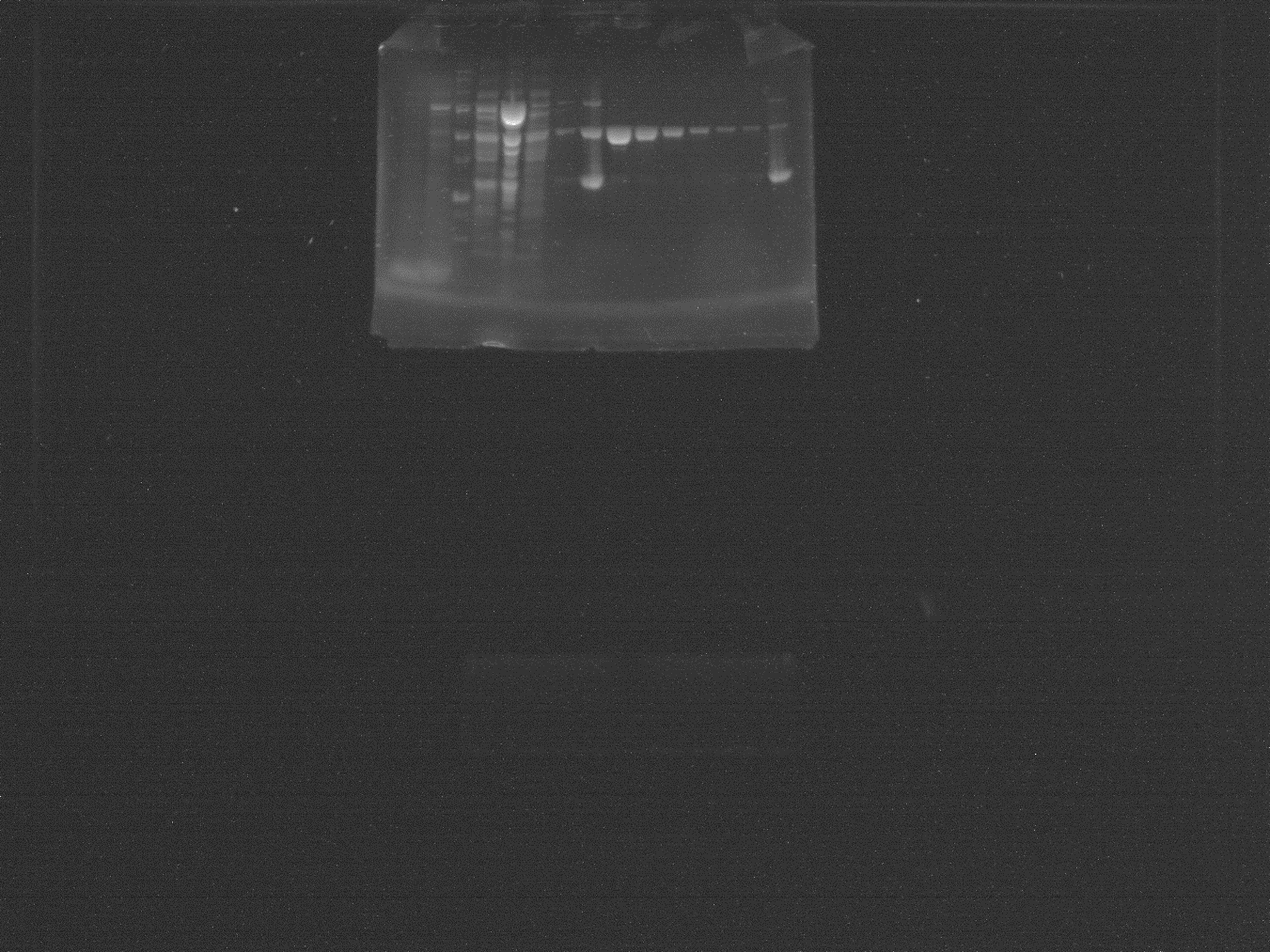


Figure 1. Raw image taken from the SDS-PAGE gel presented in the Figure 1a. The colors of the gel were inverted (color black to white) to enhance the visualization of the bands.

In addition, two intermediate lanes (A and B) and the last aliquot (C) were eliminated because the aliquots in those lanes involved the protein still embedded in the silica before separation and were not important for the presentation of the results in the manuscript.

Lane M: Molecular weight markers 250 kDa; Lane 1: cell extract before isopropyl-β-D-1-thiogalactopyranoside (IPTG) induction; Lane 2: cell extract after IPTG induction; Lane 4: cell extract pellet; Lane 5: cell extract supernatant; Lane 6: Resin before fusion protein cleavage reaction; Lanes 7-12: Elution of target protein.

**M 1 2 3 4 5 A B 6 7 8 C D E F**

**250 KDa**

**100**

**50**

**37**

**25**

**15**

**150**

**75**

**20**


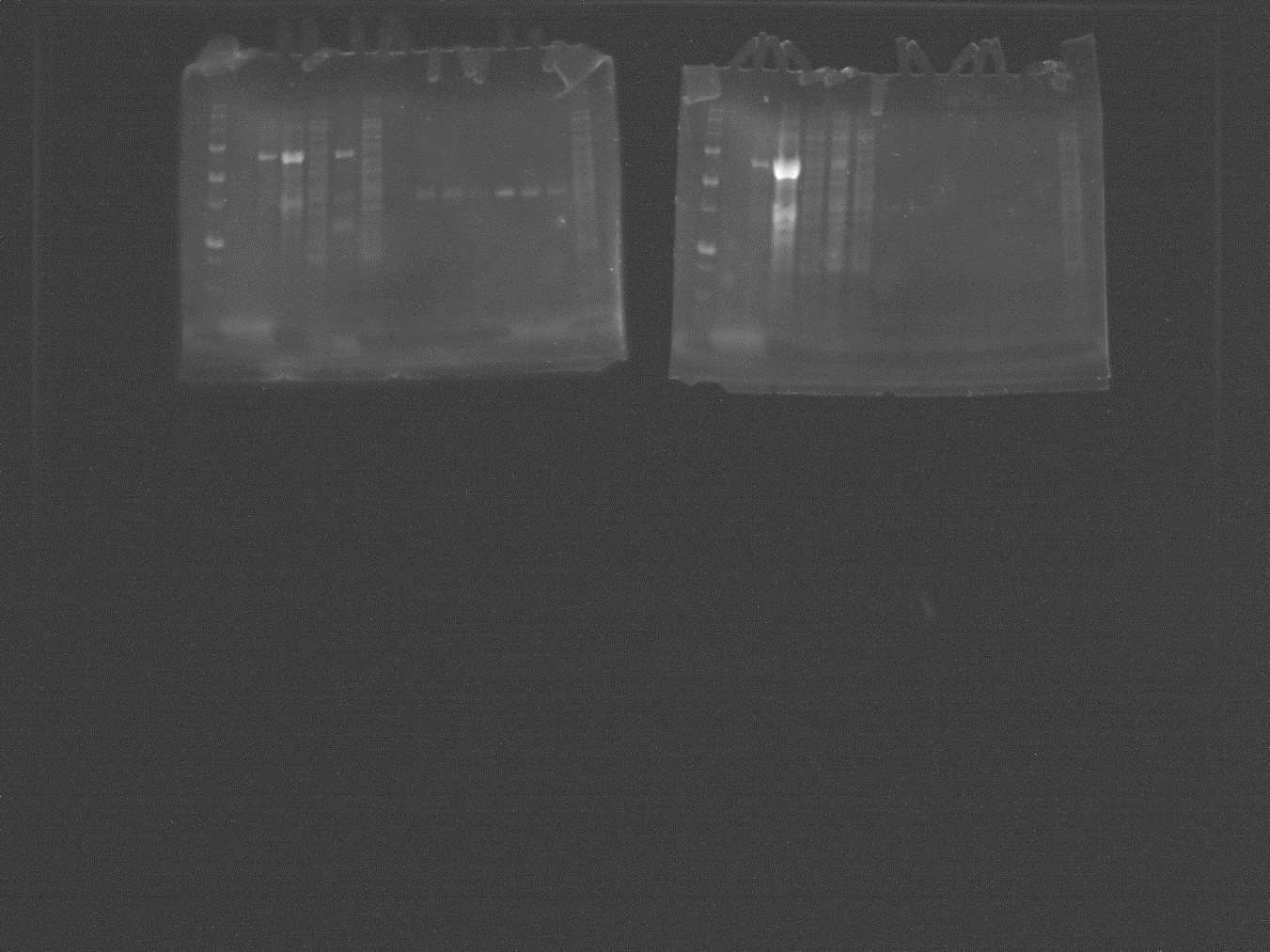


Figure 2. Raw image taken from the SDS-PAGE gel to present in the Figure 1b. The colors of the gel were inverted (color black to white) to enhance the visualization of the bands.

Also, the last 4 lanes of the gel (C, D, E and F) were cut and eliminate because we did not saw noteworthy difference after the 3 first fractions of the purified protein. Finally, two intermediate lanes (A and B) were eliminate because the first did not have enough definition and the second was empty, this can be due to an error on experimentation since those lanes involved the protein embedded in the silica before separation, moreover those aliquots were not of great importance for the presentation of the results in the manuscript.

Lane 1: cell extract before IPTG induction; Lane 2: cell extract after IPTG induction; Lane 3: cell extract pellet; Lane 4: cell extract supernatant; Lane 5: Resin before fusion protein cleavage reaction; Lanes 6-8: Elution of target protein.
